# Compost and microbial biostimulant applications improve plant growth and soil biological fertility of a grass-based phytostabilization system

**DOI:** 10.1007/s10653-022-01235-7

**Published:** 2022-03-22

**Authors:** Donato Visconti, Valeria Ventorino, Massimo Fagnano, Sheridan Lois Woo, Olimpia Pepe, Paola Adamo, Antonio Giandonato Caporale, Linda Carrino, Nunzio Fiorentino

**Affiliations:** 1 Department of Agricultural Sciences, University of Naples Federico II, 80055 Portici, Italy; 2 Department of Pharmacy, University of Naples Federico II, 80131 Naples, Italy; 3 Center for Studies on Bioinspired Agro-Environmental Technology (BAT Center), 80055 Portici, Italy; 4 Institute for Sustainable Plant Protection, National Research Council, 80055 Portici, Italy

**Keywords:** Mycorrhizae, Phytoremediation, Microbial indicators, Heavy metals, Bioaccessibility

## Abstract

In this work, a grass-based phytoremediation system integrated with an organic amendment and biostimulants was evaluated for remediating contaminated sites. Plant growth and biological fertility were monitored to assess the efficacy of a vegetative cap used as a safety measure to reduce sanitary and environmental risks of industrially contaminated soils and soil-washing sludges. Both matrices were potentially contaminated with Pb and Zn with an ecological risk index from low to moderate. According to potentially toxic elements (PTEs) bioaccessibility tests, the exposure to the released fine particulate matter may cause serious risks to human beings, in particular to children. The grass mixture was well adapted to both the substrates and a low PTEs mobility was detected, thus, reducing the leaching risk to ground water sources. Compost addition augmented significantly nitrogenase reductase (*nif*H) and ammonia monooxygenase (*amo*A) gene expression abundance in both substrates. Furthermore, a positive interaction between compost fertilization and a *Trichoderma*-based biostimulant inoculation was recorded in sludges resulting in a significant stimulation of nitrogen-fixing and ammonia-oxidizing bacteria. The application of compost and biostimulant increased soil fertility and plant growth. Furthermore, there was a slight reduction in PTE bioaccessibility, thus, improving the efficiency of the phytostabilization, limiting the resuspension and dispersion of the health-risk soil particulate.

**Supplementary Information:**

The online version contains supplementary material available at 10.1007/s10653-022-01235-7.

## Introduction

Among the most critical environmental challenges are that of pollution and the negative effects on human health, and the problem of how to resolve the elimination or removal of potentially toxic elements (PTEs) from contaminated soils can be considered as one of the most serious concerns. Potentially toxic elements can originate from smelting, mining, fertilization, and engine emissions (Sidhu, 2016). Their persistence in soils can last decades or centuries (Ghosh & Singh, 2005), threatening human health due to their tendency to accumulate in the food chain, the ingestion of the soil particulate matter, as well as for contamination of the water table (Duri et al., 2020; Sahu et al., 2012).

Traditional techniques for remediating PTEs contaminated soils (i.e., soil excavation and dumping, washing, flushing, vitrification, solidification, and incineration) are expensive and have adverse impacts on soil ecosystem services (Ali et al., 2013). An alternative remediation technique is phytoremediation, an environmental-friendly and economically viable approach (Fagnano et al., 2020a; Fiorentino et al., 2013; Ma et al., 2016). Phytoremediation of PTEs includes two main mechanisms: reduction of the contaminants by the uptake and translocation from the soil to the easily harvestable plant organs (i.e., phytoextraction,) or the stabilization of PTEs in the rhizosphere, thus, reducing the mobility of the bioavailable PTEs fraction (i.e., phytostabilization) (Fiorentino et al., 2018a; Phusantisampan et al., 2016).

The effectiveness of phytoremediation depends upon the vegetative species employed, involving the selection of plants with a high efficiency in extracting or immobilizing PTEs, as well as a proven tolerance to elevated levels of the contaminants, thus, ensuring a complete and continuous soil colonization (Carrino et al., 2020; Simon, 2005). Graminaceous species belonging to the *Poaceae* family are well known for their tolerance to PTEs stress and their suitability for phytoremediation (Visconti et al., 2018, 2019).

Frequently, plant growth and PTEs contamination may be limited to sites characterized by soils with a poor fertility (Adamo et al., 2002; Clemente et al., 2012; Ma et al., 2011). Therefore, to tackle these problems, phytoremediation can be assisted by fertilization strategies and/or inoculation with biostimulants (Fiorentino et al., 2013, 2017; Visconti et al., 2020a) able to sustain crop establishment and growth under limiting conditions. The use of organic amendments (i.e., compost) can enhance plant growth and phytostabilization capacity (Visconti et al., 2020b; Wiszniewska et al., 2016). Compost is obtained from spontaneous microbial bio-oxidation of organic matter (Alluvione et al., 2013; Spaccini et al., 2009). It is a well-known source of macro- and micronutrients, and it improves soil exposure to air, water penetration and retention, and nutrient holding capacity, thus, increasing plant growth (Fagnano et al., 2011). Furthermore, compost fertilization resulted in increased plant tolerance to PTEs as well as higher metal accumulation rates by the plants (Bernal et al., 2007; Chaiyarat et al., 2011; Walker et al., 2004).

Recently, plant beneficial microbes and biostimulant products containing *Trichoderma* and mycorrhizae have been tested in assisted phytoremediation (Fiorentino et al., 2013; Visconti et al., 2020a). Some *Trichoderma* fungal species are characterized by rapid growth and the ability to establish positive symbiotic interactions with many plant species (Zafar et al., 2007); they can enhance plant growth (Fiorentino et al., 2018b; Visconti et al., 2020a) and are tolerant to PTEs and able to enhance plant resistance and extraction ability that results in higher PTEs accumulation (Babu et al., 2014; Bareen et al., 2012). Mycorrhizae (i.e., arbuscular mycorrhizal fungi—AMF) are characterized by high affinity to a variety of plant families (Wang & Qiu, 2006), favoring an increase in water and nutrient uptake by plants (Sarathambal et al., 2017). These organisms are also capable of improving plant tolerance to PTEs by producing and releasing glomalina, a glycoprotein, in the plant rhizosphere that immobilizes metals as Cu, Cd, and Pb (Gonzalez-Chavez et al., 2004). Arbuscular mycorrhizal fungi can also adsorb PTEs to chitin in their cell walls and chelate PTEs inside the fungal cells (Makoi et al., 2009). Furthermore, AMF are also reported as promoter of plant PTEs uptake (Leung et al., 2006; Selvaraj et al., 2005).

Phytoremediation, as well as other bioremediation techniques such as compost amendment and biostimulants inoculation, are aimed at restoring soil biological fertility. In fact, soil microbial community diversity, metabolism and growth can be negatively affected by PTEs presence in the soil (Ventorino et al., 2018), thus, posing a potential risk to environmental health in terms of a reduction in microbial abundance and diversity (Fagnano et al., 2020b). Bacterial populations involved in the nitrogen cycling, such as nitrogen fixers and ammonia oxidizers, are vulnerable to any biotic and abiotic stresses and their activity is very important for the soil fertility (Pepe et al., 2013; Ventorino et al., 2007, 2016). The monitoring of microbial nitrogenase reductase (*nif*H) and ammonia monooxygenase (*amo*A) genes abundance could permit an evaluation of the soil nitrogen activity that indicates the biotic status or quality during the implementation of the phytoremediation process.

The present study aims to use methods to stimulate plant growth activity as safety measure to stabilize a contaminated industrial soil, reducing soil particulate and restoring soil biological fertility (Padmavathiamma et al., 2010; Zhao et al., 2013). The effects of microbial biostimulants and compost fertilization were evaluated on rhizosphere nitrogen cycling by bacteria, plant growth, soil covering with a grass mix, and PTE bioaccessibility. In potentially contaminated sites, the monitoring of PTE bioaccessibility may help assess the impact of risk mitigation actions such as the employment of a grass-based phytoremediation system (Khelifi et al., 2020, 2021).

## Materials and methods

### Study site description

Substrates were taken from an industrial site (300 ha) called ex-ILVA (40°48.570′N, 14°10.557′E, 2–10 m a.s.l.), situated west of Naples city (southern Italy). The site was used for steel production from 1905 to 1992 and was categorized as a National Interest Priority Site (NIPS) by the Italian Parliament in 2000 (Law388, 2000). Soils from this site had PTE concentrations (originating from industrial and volcanic activity) higher than Italian screening values (SV) for residential use; therefore, a remediation project (excavation and soil-washing) was started in 1994 by the Italian government (Adamo et al., 2002; De Vivo & Lima, 2008). Soils and sediments derived by soil washing resulted contaminated by As, Pb, and Zn with low mobility and bioavailability (Adamo et al., 2015; Rocco et al., 2018); therefore, the sanitary and environmental risks of this site can likely be due to resuspension and spread of contaminated soil particles, with drift toward the surrounding densely populated areas.

### Experimental setup

An open air mesocosm (*V*: 0.15 m^3^; *D*: 0.65 m; *H*: 0.51 m) experiment was carried out from November 2015 to November 2016 in a field at the Department of Agricultural Sciences of Naples University, Portici, NA (40°49′N, 14°21′E). Mesocosms were filled with soil (S) and post soil-washing sludge (F) obtained from the ex-ILVA brownfield.

A completely randomized design was arranged to test the effect of the following factors on growth and PTE uptake of a synthetic meadow composition made with a commercial mix of microthermal grass species (80% *Festuca arundinacea* Shreb.—tall fescue*,* 10% *Poa pratensis* L.—Kentucky bluegrass*,* 10% *Lolium perenne* L.—perennial ryegrass):

(i) 2 Substrates: pre-washing soil (S) *vs*. post-washing sludge (F);
(ii) 2 Fertilization levels: fertilization (C) with commercial green waste compost (OC: 230 g kg^−1^; TN: 8 g kg^−1^) *vs.* non-fertilized control (NoC);
(ii) 3 Biostimulant levels: TA (Panoramix, a commercial seed treatment which contained a microbial consortium of *Trichoderma* spp*.*, AMF, plant growth-promoting rhizobacteria (PGPR) (*Bacillus* spp*.*); (Koppert Biological Systems, Rotterdam, the Netherlands) *vs.* TB (Trianum-P, a commercial biofungicide containing *Trichoderma afroharzianum* (ex-*T. harzianum*) strain T22; Koppert Biological Systems, Rotterdam, the Netherlands) *vs.* a non-inoculated control (NoT).

The above-mentioned factors were fully combined to obtaining 12 treatments, arranged in triplicate, for a total of 36 mesocosms. In the fertilized experimental units, compost was mixed with the substrates (0.5% w/w corresponding to a dose of 30 Mg D.W. ha^−1^) 4 weeks before sowing. The seeds of the grass mix were surface sterilized and were coated with biostimulants (final concentration ca. 1 × 10^8^ spores ml^−1^) by applying an adequate volume of the product to cover the seed surface. Treated seeds were stirred to uniformly coat the seeds surface, air-dried then stored at 4 °C until sowing. Inoculated (TA and TB) and non-inoculated seeds were sowed on November 20th 2015 with 20 g of seeds per mesocosm. Water management was carried out during the spring–summer period to restore the available water capacity of each trial unit, and waterlogging during the rainy season was minimized providing an expanded clay drainage layer (15 cm thickness) at the bottom of each pot.

A preliminary characterization of the substrates (Table S1) showed the following features: sandy loam texture in S, silty loam in F; neutral-subalkaline pH (7.6—F; 7.5—S); low content of carbonates (3.7%—F; 5.3%—S); moderate levels of organic matter (24.8 g kg^−1^—F; 19.5 g kg^−1^—S); and total nitrogen (0.9 g kg^−1^—F; 0.7 g kg^−1^—S) with a C:N ratio of 16. According to Italian Environmental Law (Italian Law Decree 152, 2006), both substrates were potentially contaminated by As (47 mg kg^−1^—F; 41 mg kg^−1^—S), Pb (281 mg kg^−1^—F; 171 mg kg^−1^—S), and Zn (1208 mg kg^−1^—F; 362 mg kg^−1^—S) with greater values in F than in S.

### Soil and plant sampling and analysis

For the preliminary physico-chemical characterization, discrete samples of pre-washing soil and post-washing sludge were collected before sowing (October 2015) and dehydrated at 50 °C until obtaining a constant weight, homogenized, and sieved at 2 mm. The following physico-chemical properties were measured on the fraction < 2 mm: texture, pH-H_2_O (1:2.5 soil:water solution ratio) electrical conductivity (1:5 soil:water solution ratio), organic carbon (Walkley & Black method, 1934), total nitrogen (Kjeldahl, 1883), carbonate (Dietrich–Frühling calcimeter method, Loeppert & Suarez, 1996), and PTEs pseudo-total content (aqua regia digestion, ISO, 12914, 2012 followed by ICP-MS analysis at Acme Analytical Laboratories Ltd.,Vancouver, Canada). Certified reference material ERM-CC141 Loam Soil and BCR-141R Calcareous Loam Soil (European Reference Materials—ERM®) and lab standard materials (STD DS10 and STD OREAS45EA) were used to monitor the data quality of soil analyses, with metal recoveries around ± 10% of the certified values. The detection limits of As, Cd, Cu, Pb, and Zn were, respectively, 0.1, 0.01, 0.02, 0.001, and 0.025 mg kg^−1^.

Substrate samples were collected within each experimental unit with a soil sampling drill (depth: 0–30 cm) at the beginning (one month after sowing—t0) and at the termination of the experiment (t3), dried at 50 °C until constant weight, homogenized, and sieved at 2 mm. The easily available PTE content was estimated by single extraction, by using 1 M NH_4_NO_3_ (1:2.5 soil:solution ratio; DIN 19730, 1995). The PTE contents in the filtrates were measured by ICP-MS at Acme Analytical Laboratories Ltd. (Vancouver, Canada). Certified lab standard materials (STD DS12 and STD DS3) were used to monitor the data quality of soil analyses, with metal recoveries around ± 10% of the certified values. The detection limits of As, Cd, Cu, Pb, and Zn were, respectively, 0.1, 0.02, 0.02, 0.02, and 0.1 mg kg^−1^.

A standardized sampling area of 1600 cm^2^ was set up to collect grass biomass from each experimental unit on May 16th (t1—178 days after sowing, DAS), July 18th (t2—241 DAS), and November 22nd (t3—368 DAS), 2016.

Plant samples were washed (firstly with tap water and then with deionized water), dried in an oven (60 °C) until constant weight and ground before the analysis. The dry weights of the three cuttings were summed to assess the annual aboveground biomass yield. Plant samples were digested initially in a microwave oven with concentrated HNO_3_ and then with an *aqua regia* (HCl/HNO_3,_ 3:1 v:v) digestion and then analyzed by ICP-MS (analytical package VG101-EXT) at Acme Analytical Laboratories Ltd. (Vancouver, Canada) to determine PTEs contents. Certified reference material (oriental tobacco leaves CTA-OTL-1) and lab standard materials (STD CDV-1 and STD V16) were used to monitor the data quality of analyses, with metal recoveries around ± 8% of the certified values. The detection limits of As, Cd, Cu, Pb, and Zn were, respectively, 0.1, 0.01, 0.01, 0.01, and 0.1 mg kg^−1^.

Potentially toxic elements concentration in the plant samples was related to PTEs thresholds in forage (EU Reg., 1275/2013). The PTEs mean contents of grasses growing on contaminated sites reported by Kabata-Pendias (2011) were used as reference for metals not considered by this regulation.

Furthermore, with the aim to detect some nutritional stresses, the total N content of grass species was also measured (Kjeldahl, 1883).

### Quantification of bacterial functional genes

Plant roots were collected from each experimental unit at t2 (8 months after sowing) and t3 (12 months after sowing), and soil rhizosphere samples were obtained as previously described (Romano et al., 2020). Genomic DNA was extracted with the FastDNA Spin Kit for Soil (MP Biomedicals, Illkirch Cedex, France), and then 50 ng DNA was used as a template for quantitative polymerase chain reactions (qPCRs).

Polymerase chain reaction (PCR) mixture and settings were carried out as previously described (Fiorentino et al., 2016) using the primes nifH-F and nifH-R (Rösch et al., 2002) or amoA-1F and amoA-2R (Rotthauwe et al., 1997) to quantify *nif*H and *amo*A genes, respectively, by a Chrom4 System Thermocycler (Bio-Rad, Milan, Italy). The abundance of the two bacterial functional genes was calculated with a standard curve for each target gene as reported by Fiorentino et al. (2016).

### Soil covering by grasses

The soil covering by plants was considered correlated to the diminution of wind and water erosion processes as well as to PTEs immobilization in the rhizosphere (Mendez & Maier, 2008); this parameter was estimated in each mesocosm by the ratio between the vegetated area and the sampling area.

### Potential ecological risk assessment

The index recommended by Hakanson (1980), i.e., potential ecological risk index (ERI), was employed on initial soil data to assess the potential risk for biological community and bionetworks from PTEs contamination. The ERI was calculated as follows:

$$\mathrm{ERI}=\sum_{i=1}^{n}{E}_{r}^{i}=\sum_{i=1}^{n}{T}_{r}^{i}\times {C}_{f}^{i}=\sum_{i=1}^{n}\left({T}_{r}^{i}\times \frac{{C}^{i}}{{C}_{n}^{i}}\right),$$

where ${E}_{\mathrm{r}}^{\mathrm{i}}$ is the monomial potential ecological risk index of PTE *i*; ${T}_{\mathrm{r}}^{\mathrm{i}}$ is the toxic response component for a precise PTE i (e.g., As = 10, Cd = 30, Cr = 2, Cu = 5, Pb = 5, and Zn = 1); ${\mathrm{C}}_{\mathrm{f}}^{\mathrm{i}}$ is the contamination component of PTE *i*; *C*^*i*^ is the content of PTE *i* in the samples (mg kg^−1^); and ${C}_{n}^{i}$ is the PTE background value (mg kg^−1^). In this research, soil background values of the area (As: 12; Cd: 0.3 Pb: 50; Zn: 67.5 mg kg^−1^) from Cicchella et al. (2008) were used for ${C}_{n}^{i}$ parameter. In particular, the background values used in this work were derived from composite samples (*n* = 982) from Naples provincial area. Potentially toxic elements’ soil concentrations were elaborated using the GeoDas program (Cicchella et al., 2008).

The monomial potential ecological risk of each PTE (${E}_{r}^{i}$) was considered as low (${E}_{r}^{i}$< 40), moderate (40 ≤ ${E}_{r}^{i}$<80), considerable (80 ≤ ${E}_{r}^{i}$<160), high (160 ≤ ${E}_{r}^{i}$<320), and very high (${E}_{r}^{i}$ ≥ 320). The aggregate risk represented by ERI was classified as low (ERI < 95), moderate (95 ≤ ERI < 190), high (190 ≤ ERI < 380) , and very high (ERI ≥ 380) (Rehman et al., 2018).

### In vitro* oral, inhalation, and dermal bioaccessibility tests and health-risk assessment*

The oral, inhalation, and dermal bioaccessibility of As, Cd, Pb, and Zn in designated soil (S) and sludge (F) samples were assessed by (i) Simple Bioaccessibility Extraction Test (SBET; Oomen et al., 2002; U.S.EPA, 2017: Method 1340), simulating the harsh and acid environment of stomach (pH 1.5); (ii) Artificial Lysosomal Fluid (ALF), mimicking the acid lysosomal fluid (pH 4.5) secreted by alveolar macrophages (Stopford et al., 2003) in the pulmonary alveoli to engulf particulate matter within several hours afterward deposition; (iii) Simulated Skin Surface Liquid (NIHS 96-10; Leal Chaparro et al., 2018), simulating the acid (pH 4.7) human sweat produced by the eccrine sweat glands.

Medium-fine particle-size fractions (< 2, 2–10, 10–20 and 20–50 µm) were physically separated from the coarser particles (> 50 µm) of the following soil (S) and sludge (F) samples: non-fertilized and non-inoculated treatment (NoC/NoT; t0: before, t3: after, plant growth, respectively), and compost-fertilized + microbial consortium treatment (t3: after plant growth), in accordance with the protocol described by Khelifi et al. (2021).

Each medium-fine particle-size fraction (< 2, 2–10, 10–20, and 20–50 µm) was extracted by SBET, ALF, and NIHS 96-10 analytical procedures in a thermostatic water bath at 37 ± 1 °C under vibration (80 oscillations min^−1^), with a solid/solution ratio of: 1:100 (SBET), 1:50 (ALF), and 1:25 (NIHS 96-10), for 1 h (SBET), 24 h (ALF), and 8 h (NIHS 96-10) on the basis of physiological residence time or exposure duration (Khelifi et al., 2021). Afterward, extracts were centrifuged, filtered, acidified, and kept at 4 °C until analysis by Inductively Coupled Plasma—Optical Emission Spectrometry (ICP-OES, Thermo Scientific iCAP 7400). Instrument calibration and analytical quality checks were performed through blanks and standards containing known concentrations of PTEs. The detection limits were 0.1 mg kg^−1^ (Cd), 0.2 mg kg^−1^ (Zn), and 0.5 mg kg^−1^ (As and Pb).

The bioaccessible contents of PTEs extracted from the finest particle-size fractions (< 2 and 2–10 µm) of NoC/NoT—t0 soil (S) and sludge (F) samples were used in a US.EPA-based risk assessment assay (US.EPA, 2011), to quantify the non-carcinogenic (NCR) and carcinogenic (CR) risks for human health. These risks are related to the oral, inhalation, and dermal exposure to bioaccessible contents of PTEs, which can arise from the suspension of S and F particulate matter in the air, if not adequately protected by a green capping. The parameters and reference values applied in the risk assessment of this study are widely described elsewhere, in Khelifi et al. (2021).

### Statistical analysis

The statistical analyses were performed by using Ms Excel 2013 and SPSS 21 (SPSS Inc. Chicago, USA). Analysis of variance (ANOVA) was executed by using a general linear model. Means were separated according to LSD test (*p* < 0.05). Kolmogorov–Smirnov and Levene tests were carried out to attest normality of distribution and homogeneity of variance, respectively. Logarithmic transformation was applied to studied variables, when necessary, to ensure normality of distribution.

## Results

### Ecological risk assessment of the substrates

Main factor effects on ${E}_{r}^{i}$ (As, Cd, Pb, and Zn) and ERI are shown in Table S2.

Only the substrate average effect was statistically significant. F reported higher As, Cd, Pb, and Zn concentration as well as monomial risks (${E}_{r}^{i}$) respect to S. The monomial risk of As, Cd, Pb, and Zn was low (${E}_{r}^{i}$< 40) except for Cd in F that was moderate (${E}_{r}^{i}$ = 65). However, the combined risk represented by ERI was moderate in F (${E}_{r}^{i}$ = 148) and low in S (${\mathrm{E}}_{\mathrm{r}}^{\mathrm{i}}$ = 89).

### Plant biomass, nitrogen uptake, and soil covering

**Fig. 1:**
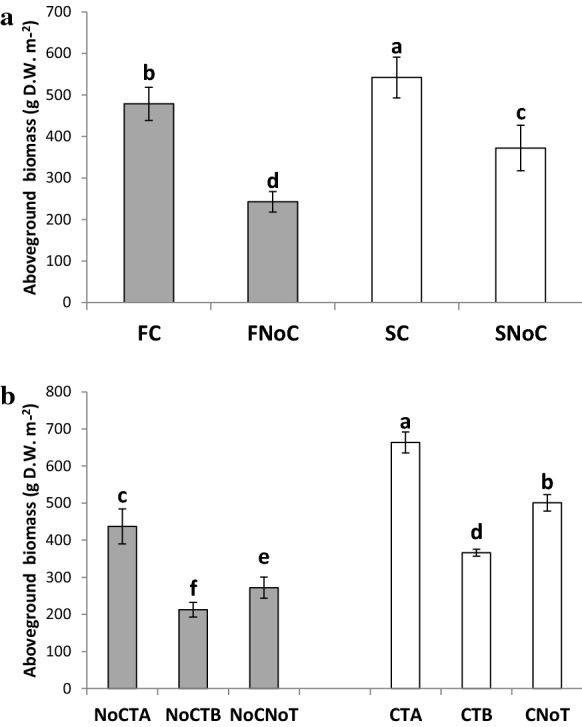
Fertilization and biostimulant effect on biomass production. **a** Fertilization by substrate interaction; **b** Compost by biostimulant interaction. C and NoC are compost and non-fertilized treatments, respectively; TA, TB, and NoT are a microbial consortium, *Trichoderma harzianum* T22, and non-inoculated treatments, respectively. F and S are soil-washing sludge and pre-washing soil. Bars indicate ± standard errors. Mean values with the same letter do not differ according to the LSD test (*p* < 0.05)

Main factor effects on annual cumulative biomass production, nitrogen content, nitrogen uptake, and soil covering are shown in Table 1. The interaction between substrate and fertilization (Sub × Fert) was significant for plant growth (Fig. 1a), nitrogen content, and uptake (Table 2) while a compost by biostimulant interaction (Fert × Bio) was observed for biomass production (Fig. 1b) and nitrogen uptake (Table 2). On the average, grass species grown on sludge (F) showed a lower biomass accumulation (− 23%) than on soil (S), while compost application increased plant growth (+ 66%), nitrogen uptake (+ 67%), and soil covering (+ 3%) (Table 1). Biostimulants showed different effects: TA significantly increased growth (+ 42%), nitrogen uptake (+ 32%) ,and soil covering (+ 2%) as compared to NoT, although a lower nitrogen content was recorded, while the same variables were significantly lower than the control with TB (− 25%, − 30%, and − 2% for biomass, nitrogen uptake, and soil covering, respectively) (Table 1).

**Table 1:** Main factor effects on cumulative biomass production, average nitrogen content, cumulative nitrogen uptake, and soil covering

Treatments	Aboveground biomass (g D W m^−2^)	*N* (%)	*N* (mg m^−2^)	Soil covering (%)
Substrate				
F	360**b**	2.43	887**b**	94
S	456**a**	2.39	1077**a**	96
Fertilization				
C	510**a**	2.42	1230**a**	97**a**
NoC	307**b**	2.39	734**b**	94**b**
Biostimulants				
TA	550**a**	2.32**b**	1284**a**	97**a**
TB	289**c**	2.34**b**	686**c**	93**c**
NoT	386**b**	2.56**a**	975**b**	95**b**
Mean	408	2.41	982	95
*Significance*				
Sub	**	*n.s*	**	*n.s*
Fert	**	*n.s*	**	*
Bio	**	*	**	*
Sub × Fert	**	*	**	*n.s*
Sub × Bio	*n.s*	*n.s*	*n.s*	*n.s*
Fert × Bio	*	*n.s*	*	*n.s*
Sub × Fert x Bio	*n.s*	*n.s*	*n.s*	*n.s*

Substrates F and S are the soil-washing sludge and pre-washing soil, respectively. Fertilization treatments are C and NoC of compost fertilized and non-fertilized, respectively; Biostimulants are TA, TB, and NoT containing a microbial consortium, *Trichoderma* T22, and non-inoculated treatments, respectively. Sub is substrate (F vs S) factor; Fert is fertilization (C s NoC) factor; Bio is biostimulant (TA vs TB vs NoT) factor. Mean values with the same letter do not differ according to the LSD test (*p* < 0.05). *p < 0.05; **p < 0.01; *n.s*, not significant

**Table 2:** Compost and biostimulant effect on the average N content and cumulative N uptake

Treatments	*N* (%)	*N* (mg m^−2^)
Interaction Sub × Fert		
FC	2.53**a**	1207**b**
FNoC	2.33**b**	567**d**
SC	2.32**b**	1253**a**
SNoC	2.46**a**	902**c**
Mean	2.41	982
Interaction Fert × Bio		
NoCTA	2.26	988**c**
NoCTB	2.27	490**f**
NoCNoT	2.66	724**e**
CTA	2.39	1579**a**
CTB	2.41	883**d**
CNoT	2.47	1227**b**
Mean	2.41	9824

C and NoC are compost-fertilized and non-fertilized treatments, respectively; TA, TB, and NoT are a microbial consortium, *Trichoderma* T22, and non-inoculated treatments, respectively. F and S are soil-washing sludge and pre-washing soil. Sub is substrate (F vs S) factor; Fert is fertilization (C s NoC) factor; Bio is biostimulant (TA vs TB vs NoT) factor. Mean values with the same letter do not differ according to the LSD test (*p* < 0.05)

Compost fertilization positively affected meadow growth (Fig. 1a), with a 97% and 46% increase in F and S, respectively, and N uptake (Table 2) with a 113% and 39% increase for F and S, respectively.

In the absence of compost fertilization, only TA application increased grass growth (Fig. 1b), with a 60% increase, and N uptake (Table 2), with a 37% increase than in the non-inoculated mesocosms. The concomitant application of compost and biostimulant did not change this pattern even if the increase obtained with TA was lower than in the unfertilized mesocosms (+ 32% and + 29% for biomass and N uptake, respectively) (Table 2; Fig. 1b). The application of TB with or without compost (CTB and NoCTB) was associated to a decrease of both grass growth (− 24% on the average) and N uptake (− 30% on the average) compared to the non-inoculated control (Table 2; Fig. 1b).

### Zinc and Lead uptake and bioavailability

Compost fertilization increased Pb concentration in plants while an opposite effect was recorded for Zn (Table 3). No effect of biostimulants was recorded on plant uptake of both PTEs while the substrate by fertilization interaction on Pb content was significant (Fig. S1). Lead concentration in shoots was higher in FNoC than in SNoC (Fig. S1), while compost fertilization in S substrate (Fig. S1) was associated to the highest Pb content.

**Table 3:** Main factors effects on the average Pb and Zn content in aboveground tissues

Treatments	Pb (mg kg^−1^)	Zn (mg kg^−1^)
Substrate		
F	0.68	70**a**
S	0.74	38**b**
Fertilization		
C	0.81**a**	51**b**
NoC	0.61**b**	57**a**
Biostimulants		
TA	0.66	53
TB	0.74	55
NoT	0.74	54
Mean	0.71	54
*Significance*		
Sub	n.s	**
Fert	**	**
Bio	n.s	n.s
Sub × Fert	**	n.s
Sub × Bio	n.s	n.s
Fert × Bio	n.s	n.s
Sub × Fert × Bio	n.s	n.s

C and NoC are compost-fertilized and non-fertilized treatments, respectively; TA, TB, and NoT are a microbial consortium, *Trichoderma* T22, and non-inoculated treatments, respectively. F and S are soil-washing sludge and pre-washing soil. Sub is substrate (F vs S) factor; Fert is fertilization (C s NoC) factor; Bio is biostimulant (TA vs TB vs NoT) factor. Mean values with the same letter do not differ according to the LSD test (*p* < 0.05). *p < 0.05; **p < 0.01; *n.s.* not significant

A very low easily available (NH_4_NO_3_ extractable) Pb and Zn concentrations were detected in S substrate, and they were under the limits of detection (Pb lower than 0.02 mg kg^−1^ and Zn lower than 0.1 mg kg^−1^), while values recorded for F after crop establishment at both t1 (0.05 mg Pb kg^−1^; 0.55 mg Zn kg^−1^) and t3 (0.05 mg Pb kg^−1^; 0.56 mg Zn kg^−1^) did not show differences (data not shown).

### Soil microbial fertility, PTE bioaccessibility, and health-risk assessment

**Fig. 2:**
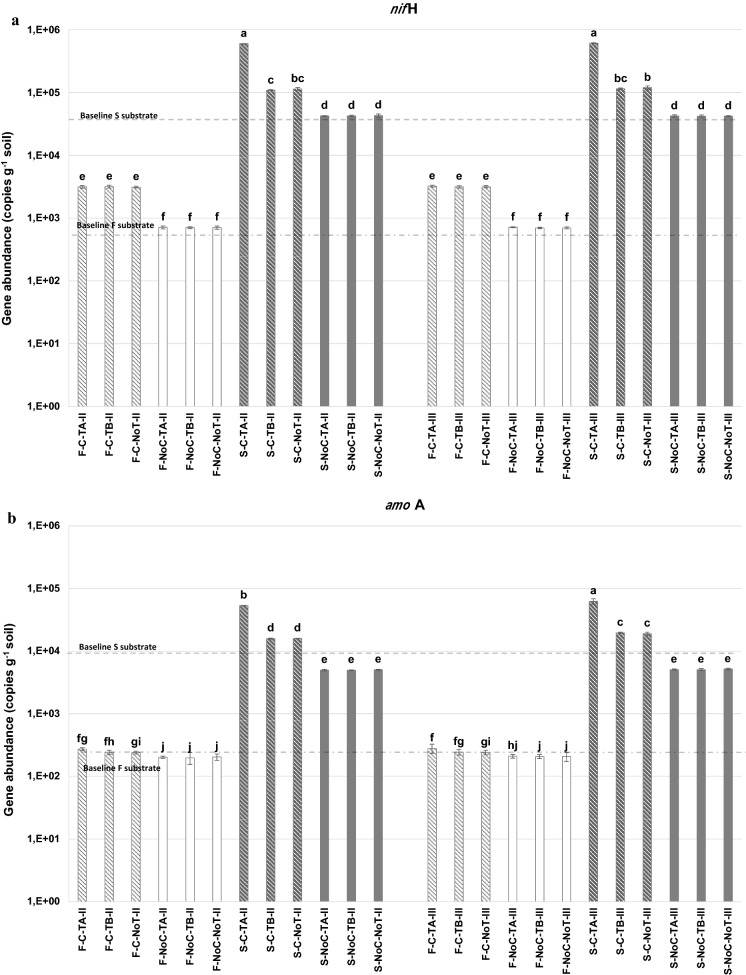
Abundance of gene copies per g of soil as determined by qPCR for the *nif*H gene (**a**) and *amo*A gene (**b**) determined at different sampling times, t2 = II (8 months after sowing) and t3 = III (12 months after sowing) in rhizo compartments of pre-washing soil (S) and soil-washing sludge (F) substrates. C and NoC are compost and non-fertilized treatments, respectively; TA, TB, and NoT are a microbial consortium, *Trichoderma afroharzianum* T22, and non-inoculated treatments, respectively. Dashed lines represent the baseline values measured of the F and S substrates before meadow sowing at t1. Mean values with different letters are statistically significantly different (*p* < 0.05). Bars indicate ± standard errors (*n* = 3)

Statistically significant differences were recorded by comparing N_2_-fixing and nitrifying population size of rhizo-soils, reporting a higher *nif*H and *amo*A genes abundance in S than in F unrelatedly of sampling time and treatments (Fig. 2).

In both substrates (F and S), compost fertilization led to a considerable increase of the *nif*H gene abundance as compared to the values recorded in the same substrate at the beginning of the experiment before microthermal grass plants sowing (4.7 × 10^2^ copies g^−1^ substrate and 4.8 × 10^4^ copies g^−1^ substrate in F and S, respectively) (Fig. 2a), whereas the values remained almost constant in non-amended soils (Fig. 2a). Moreover, the *nif*H gene abundance was impacted by a compost and biostimulant interaction in S substrate. In fact, a higher significant abundance in gene copies was observed in SC (1–6 × 10^5^ copies g^−1^ substrate) in comparison to SNoC (4.2–4.3 × 10^4^ copies g^−1^ substrate) at both sampling times (Fig. 2a). Among the SC samples, the biostimulant applications greatly influenced diazotrophic populations depending upon the type of microorganism contained (microbial consortium vs *Trichoderma* alone), whereby TA significantly increased the abundance of *nif*H copies at both sampling times (5.9–6.1 × 10^5^ copies g^−1^ substrate) when compared to the control NoT (1.1–1.2 × 10^5^ copies g^−1^ substrate) and TB (1.0–1.1 × 10^5^ copies g^−1^ substrate) which were similar (Fig. 2a).

Conversely, no inoculation effect was detected on diazotrophic populations in SNoC since no significant differences in the *nif*H gene abundance were detected following the treatments with biostimulants (S-NoC-TA and S-NoC-TB) in respect to the control (S-NoC-NoT) over the sampling times (t2 and t3) (Fig. 2a).

Compost fertilization exerted a similar effect on N_2_-fixing populations in the F substrate (Fig. 2a) resulting in a higher abundance of *nif*H gene (about 3.1 × 10^3^ copies g^−1^ substrate and 7.0 × 10^2^ copies g^−1^ in FC than FNoC, respectively), while no effect was recorded with biostimulant applications, neither in the compost treated and non-treated soils (FC and FNoC).

In S substrates amended with compost (SC), the *amo*A gene abundance was considerably higher over time, over that recorded at the beginning of the experiment (8.6 × 10^3^ copies g^−1^ substrate), while in the non-amended soil (SNoC), the level of this gene remained lower and unvaried overtime (Fig. 2b). Interestingly, in F substrates, the *amo*A gene abundance was only slightly higher than baseline values (2.4 × 10^2^ copies g^−1^ substrate) regardless of sampling time and treatments (Fig. 2b).

Compost fertilization and biostimulant applications positively affected the population of ammonia oxidizers in S but only marginally in the F substrates (Fig. 2b). Indeed, the abundance of *amo*A gene in F substrate remained almost constant during the experiment (about 2–2.8 × 10^2^ copies g^−1^ substrate) even if a slight but significant increase was recorded on fertilized soils. In S substrate, although no differences were detected among NoC soils, where the *amo*A gene was relatively stable ranging from 4.2 × 10^4^ to 4.3 × 10^4^ copies g^−1^ substrate, a highly significant increase was observed in soils treated with the compost (Fig. 2b). Above all, the highest *amo*A gene copies abundance was detected in the TA-treated soils in both sampling times (about 6.0 × 10^5^ copies g^−1^ substrate) over the TB and NoT. The effect of compost and biostimulant applications in S substrate was more marked at t3 where there was an increase in all soils.

The oral and inhalation bioaccessible contents of extracted PTEs (particle size: < 2, 2–10, 10–20, and 20–50 µm) of S and F samples were significantly higher than the dermal bioaccessible contents (Table 4, line: Mean).

**Table 4:** Oral, inhalation, and dermal bioaccessible contents (mg kg^−1^) of potentially toxic elements (PTEs) extracted from medium-fine particle-size fractions (< 2, 2–10, 10–20, and 20–50 µm) of soil (S) and sludge (F) samples by SELF, ALF, and NIHS analytical procedures

Treatments	SBET (oral bioaccessible contents)				ALF (inhalation bioaccessible contents)				NIHS (dermal bioaccessible contents)			
	As	Cd	Pb	Zn	As	Cd	Pb	Zn	As	Cd	Pb	Zn
	(mg kg^−1^)				(mg kg^−1^)				(mg kg^−1^)			
Substrate (Sub)												
F	13	2.1**a**	192**a**	1230**a**	14	1.9**a**	130**a**	1168**a**	3.9	0.8**a**	3.2**a**	101**a**
S	11	0.8**b**	99**b**	267**b**	12	0.7**b**	65**b**	264**b**	3.9	0.4**b**	2.2**b**	28**b**
Plant-BioF (PBF)												
C + TA—t3	12	1.4	143	734	13	1.2	93	657	3.8	0.6	2.8	64
NoC/NoT—t3	12	1.5	147	759	12	1.3	95	685	3.9	0.6	2.7	64
NoC/NoT—t0	12	1.5	147	753	14	1.5	105	805	4.0	0.6	2.6	66
Particle-size (PS)												
2 µm	18**a**	2.8**a**	283**a**	1552**a**	23**a**	2.7**a**	176**a**	1507**a**	4.2**a**	0.9**a**	0.9**c**	41**b**
2–10 µm	14**b**	1.6**ab**	163**b**	796**ab**	13**b**	1.4**ab**	108**b**	703**ab**	3.3**b**	0.6**b**	1.0**c**	35**b**
10–20 µm	9.3**c**	0.8**b**	74**c**	347**b**	8.5**c**	0.7**b**	57**c**	350**b**	4.1**a**	0.4**b**	3.9**b**	90**a**
20–50 µm	8.0**c**	0.7**b**	62**c**	300**b**	7.8**c**	0.6**b**	49**c**	302**b**	4.0**a**	0.4**b**	5.0**a**	93**a**
Mean	12**A**	1.5**A**	146**A**	749**A**	13**A**	1.3**A**	98**B**	716**A**	3.9**B**	0.6**B**	2.7**C**	65**B**
*Significance*												
Sub	*n.s*	**	*	**	*n.s*	**	*	**	*n.s*	**	*	**
BioF	*n.s*	*n.s*	*n.s*	*n.s*	*n.s*	*n.s*	*n.s*	*n.s*	*n.s*	*n.s*	*n.s*	*n.s*
PS	*	*	**	**	*	*	*	**	*	*	**	*
Sub × BioF	*n.s*	*n.s*	*n.s*	*	*n.s*	*n.s*	*n.s*	*	*n.s*	*n.s*	*n.s*	*n.s*
Sub × PS	*n.s*	*	*	**	*n.s*	*	*	**	*n.s*	*	*	*
BioF × PS	*n.s*	*n.s*	*n.s*	*	*n.s*	*n.s*	*n.s*	*	*n.s*	*n.s*	*n.s*	*n.s*
Sub × BioF × PS	*n.s*	*n.s*	*n.s*	*n.s*	*n.s*	*n.s*	*n.s*	*n.s*	*n.s*	*n.s*	*n.s*	*n.s*

SBET is the acronym of Simple Bioaccessibility Extraction Test and ALF of Artificial Lysosomal Fluid, NIHS refer to Simulated Skin Surface Liquid. C + TA indicates compost-fertilized + microbial consortium treatment (t3: after plant growth), NoC/NoT refers to non-fertilized and non-inoculated treatment (t0: before, t3: after, plant growth, respectively). F and S are soil-washing sludge and pre-washing soil. Sub is substrate (F vs S) factor; PBF is plant and (bio)fertilization (C + TA—t3 vs NoC/NoT—t3 vs NoC/NoT—t0) factor; PS is particle-size (< 2 vs 2–10 vs 10–20 vs 20–50 µm) factor. Mean values with the same letter do not differ according to the LSD test (*p* < 0.05). *p < 0.05; **p < 0.01; *n.s.* not significant

Significantly higher bioaccessible PTE fractions (except As) were extracted from medium-fine particles of F than S samples (Table 4), in agreement with the different pseudo-total contents (Table S1).

The fine particle-size fractions (< 2.5 and 2.5–10 µm) showed the higher PTE bioaccessible fractions as compared to the coarse particle-size fractions (10–20 and 20–50 µm; up to fivefold: Table 4). On the other hand, a slight reduction (although not statistically significant) of the PTE bioaccessibility due to effect of plant growth (NoC/NoT—t0 vs NoC/NoT—t3: − 4.5% on average), or plant growth combined to substrate (bio)-fertilization (NoC/NoT—t0 vs C + TA—t3: − 6.2% on average), in particular of the inhalation bioaccessibility.

The US.EPA-based risk assessment (Table 5) revealed that the exposure to oral, inhalation, and dermal bioaccessible contents of PTEs extracted from fine (< 2 and 2–10 µm) particles of NoC/NoT S and F samples could cause serious NCR (Hazard Index: HI > 1 for children) and CR (> 10^–4^) risks for human health, especially in children. These risks are mostly due to the ingestion route and to PTEs such as As and Pb (Table 5).

**Table 5:** Non-carcinogenic (NCR: top) and carcinogenic (CR: down) human health risks due to ingestion (OR), inhalation (IN), and dermal contact (DE) of/with bioaccessible fractions of PTE extracted from medium-fine particle-size fractions (< 10 µm) of soil (S) and sludge (F) non-(bio)fertilized (NoC/NoT) samples

Substrates	Exposure routes	Adult					Children				
		HQ As	HQ Cd	HQ Pb	HQ Zn	**HI PTE**	HQ As	HQ Cd	HQ Pb	HQ Zn	**HI PTE**
S	OR	6.5E−02	1.5E−03	5.6E−02	1.7E−03	**1.2E−01**	6.1E−01	1.4E−02	5.2E−01	1.6E−02	**1.2E+00**
S	IN	1.6E−05	1.3E−07	3.3E−06	1.5E−07	**2.0E−05**	4.5E−05	3.6E−07	9.3E−06	4.3E−07	**5.5E−05**
S	DE	1.8E−04	2.8E−04	8.7E−06	1.9E−06	**4.7E−04**	1.2E−03	1.8E−03	5.7E−05	1.2E−05	**3.1E−03**
**S**	**OR + IN + DE**	**6.5E−02**	**1.8E−03**	**5.6E−02**	**1.7E−03**	**1.2E−01**	**6.1E−01**	**1.6E−02**	**5.2E−01**	**1.6E−02**	**1.2E+00**
F	OR	6.7E−02	3.6E−03	9.4E−02	7.1E−03	**1.7E−01**	6.3E−01	3.4E−02	8.8E−01	6.6E−02	**1.6E+00**
F	IN	1.9E−05	3.6E−07	6.4E−06	7.0E−07	**2.7E−05**	5.3E−05	9.9E−07	1.8E−05	1.9E−06	**7.4E−05**
F	DE	1.4E−04	5.1E−04	8.8E−06	4.7E−06	**6.7E−04**	9.4E−04	3.3E−03	5.8E−05	3.1E−05	**4.4E−03**
**F**	**OR + IN + DE**	**6.7E−02**	**4.2E−03**	**9.4E−02**	**7.1E−03**	**1.7E−01**	**6.3E−01**	**3.7E−02**	**8.8E−01**	**6.6E−02**	**1.6E+00**
		CR As	CR Cd	CR Pb	CR Zn	**CR PTE**	CR As	CR Cd	CR Pb	CR Zn	**CR PTE**
S	OR	1.3E−05	4.0E−06	2.3E−02	–	**2.3E−02**	1.2E−04	3.8E−05	2.2E−01	–	**2.2E−01**
S	IN	1.3E−10	2.0E−11	2.8E−07	–	**2.8E−07**	3.7E−10	5.7E−11	7.7E−07	–	**7.7E−07**
S	DE	6.1E−09	–	–	–	**6.1E−09**	4.0E−08	–	–	–	**4.0E−08**
**S**	**OR + IN + DE**	**1.3E−05**	**4.0E−06**	**2.3E−02**	–	**2.3E−02**	**1.2E−04**	**3.8E−05**	**2.2E−01**	–	**2.2E−01**
F	OR	1.3E−05	9.6E−06	3.9E−02	–	**3.9E−02**	1.3E−04	9.0E−05	3.6E−01	–	**3.6E−01**
F	IN	1.6E−10	5.7E−11	5.4E−07	–	**5.4E−07**	4.3E−10	1.6E−10	1.5E−06	–	**1.5E−06**
F	DE	4.8E−09	–	–	–	**4.8E−09**	3.2E−08	–	–	–	**3.2E−08**
**F**	**OR + IN + DE**	**1.3E−05**	**9.6E−06**	**3.9E−02**	–	**3.9E−02**	**1.3E−04**	**9.0E−05**	**3.6E−01**	–	**3.6E−01**

HQ stands for Hazard Quotient and HI indicates Hazard Index (i.e., the sum of HQ of all the PTE)

## Discussion

### Compost fertilization and biostimulants application promote meadow growth on industrial sediments

Plant soil covering was significantly increased by compost fertilization up to 97% of the soil surface (Table 1). Nevertheless, values ranged between 94 and 96%, indicating that tested substrates were not limiting the growth of grasses even in the absence of treatments. It is well known that an adequate vegetative capping can counteract wind and water erosion, avoiding dispersion of potentially contaminated soil particles. According to Loch (2000), a 47% vegetation cover can lower water erosion in mining areas from 30 to 35 Mg ha^1^ to 0.5 Mg ha^1^. This is because a well consolidated turfgrass can increase soil strength up to 6–18 kPa, thus, reducing soil erosion (Simon & Collison, 2002). Studies carried out by Lee et al. (2020) showed a significant effect of vegetation cover on wind erosion of sand dunes. According to the authors, when a wind blows on dunes at a wind speed of 10.8 m s^−1^, a vegetation covering of 80% of the surface reduced the erosion rate by 90% up to 98%, (corresponding to an erosion rate of 12.6 g m^2^ s^−1^ for a non-covered dune soil). According to these results, the grass mix tested in our experiment can ensure the phytostabilization and securing of the site when soil fertility is properly managed, producing a significant reduction of the particulate lift. In addition, a dense turfgrass can minimize soil disturbance due to tillage, allowing a significant increase of soil organic matter in the mid-long period especially in fine-textured soils (Forte et al., 2017).

Overall, plant growth (Table 1) was negatively affected by F substrate, with an average cumulative aboveground biomass of 360 g DW m^2^ compared to 456 g DW m^2^ of S substrate. Values recorded in S substrate were consistent with those reported by Song et al. (2015), for an 8-year turfgrass (kentucky bluegrass—*Poa pratensis* cv. Baron) stand grown on non-contaminated soils and mowed to a height of 3.8 cm (almost 420 g DW m^2^).

On the average, the addition of compost and TA increased plant biomass values up to 510–550 g DW m^2^, indicating that both crop management strategies can be adopted to overcome substrate growth limiting conditions and also permit an efficient vegetative cap development due to a dense and persistent turfgrass cover. This was clear for both substrates since the application of compost increased the plant growth (Fig. 1 a) by 97% and 46% in F and S substrates, respectively. Madejón et al. (2006) in mine soils and Ulriksen et al. (2012) in industrial soils reported a similar biomass and soil covering increase due to organic fertilization.

Biostimulants like *Trichoderma* may positively modify the rhizosphere microbial composition and increase soil nutrient solubilization and uptake by plants, thus, enhancing plant growth (Harman, 2000; Harman et al., 2004a, b). Moreover, Wei Lin and Zhang (2006) reported that *Trichoderma harzianum* can synthetize plant hormone-like growth promoters such as gibberellic acid and indoleacetic acid. However, the application of *T. afroharzianum* alone in our experiment was not associated to positive outcomes since TB caused a reduction of plant growth and soil covering (Tables 1, 2). Negative effects on plant growth have been previously reported by Harman (2006) for T22 applied to other *Poaceae* species. In maize, this was attributed to a strong genetic component that affects the plant response to T22, thus, causing a high variability in plant growth noted in decreased yield. For this reason, a better knowledge of *Trichoderma,* plant, and soil properties interactions is needed to understand how different strains and crop genotypes can be combined to produce less variable results in the field.

Nitrogen concentration was positively affected by compost fertilization only in F, while an opposite trend was found in S with a 6% reduction in fertilized mesocosms (Table 2), probably due to the dilution effect of the increased biomass production (Riedell, 2010; Baiyi et al., 2018; Ma & Zeng, 2018). The different growth and N content of plants in the two substrates can be explained by the differences in their fertility as already reported by Fiorentino et al. (2017). The authors recorded a lower nutritional stress for plants grown in S compared to F highlighting that this was coherent with a higher abundance of nitrogen cycle bacteria in S than in F substrate in the absence of soil amendment.

The higher nitrogen uptake in compost-fertilized treatments was in accordance with previous studies (Liu et al., 2016; Moreno-Jimenez et al., 2016; Singh et al., 2010), which indicated that organic fertilization promotes crop growth and nitrogen uptake, improving soil microbial activity and increasing soil organic matter pool. Compost can allow a long-term reserve of nutrients to soil that can be released slowly to plants depending on availability and mineralization rates (Fiorentino et al., 2016; Rosen & Allan, 2007; Sullivan et al., 2002).

The co-application of the microbial consortium containing *Trichoderma* spp., mycorrhiza, PGPR, and humic acids, noted with the CTA treatment, was a more successful strategy that resulted in increased biomass production (Fig. 1b) and nitrogen uptake (Table 2). These findings demonstrated that growth promotion of the meadows grass mixture in soils with poor chemical, physical, and biological features requires a synergy between compost and microbial biostimulants like *Trichoderma*, mycorrhizal fungi and nitrogen-fixing bacteria. It is well known that mycorrhiza can enhance plant tolerance to stress, and by colonizing the root surface area plus the surrounding soil zone, these symbionts increased plant growth and nutrient uptake in contaminated soils (Leung et al., 2013).

A synergic effect on plant biomass production and nitrogen uptake was reported with compost and TA application (Fig. 1b; Table 2) being consistent with findings by Vasconez et al. (2017) that reported a higher biomass production by *Lolium perenne* L. (perennial ryegrass) with combining compost and *Trichoderma* inoculation. Results were consistent with findings of Wu et al. (2011) in Pb/Zn contaminated tailings using a combination of compost and mycorrhizae that reduced the PTE toxicity increasing plant biomass and nitrogen uptake.

### Influence of compost fertilization on PTE transfer from soil to plants

Phyto-availability of Pb and Zn, represented by soil ammonium-nitrate extractable fraction, was very low for F substrate (average values of 0.05 and 0.55 mg kg^−1^ for Pb and Zn, respectively) and below the detection limits for S suggesting a low PTEs availability to plants (data not shown).

Lead content in shoots in the absence of fertilization was higher in F due to the higher pseudo-total and extractable fraction of Pb in this substrate with respect to S (in which the extractable Pb content was not detectable) (Fig. S1). Compost fertilization was associated to an increase in plant Pb content only in S substrate (Fig. S1) in accordance with Padmavathiamma and Li (2010) who stated a higher Pb uptake by perennial ryegrass, creeping red fescue, and Kentucky bluegrass after compost application in Pb/Mn-contaminated soils. Nevertheless, Pb concentration in the aboveground tissues of turfgrass (0.64 mg kg^−1^) was lower than values reported in plants growing in non-contaminated sites (2.09 mg Pb kg^−1^—Kabata-Pendias, 2011) as well as PTE thresholds in forage (34 mg Pb kg^−1^—EU Reg., 1275/2013). This result indicates that, even when pseudo-total Pb values are above the limit of screening values, grass-based phytoremediation systems do not represent a risk for the trophic chain in the event of accidental grazing or in presence of wild fauna.

Plants’ Zn concentration was higher than values reported in plants growing in non-contaminated sites (31.5 mg kg^−1^—Kabata-Pendias, 2011) (Table 3). In addition, there was a higher Zn concentration in plants grown on F than on S in all the harvests (mean value: 80 mg kg^−1^)_,_ in accordance with values reported by Zhao et al. (2013) in *F. arundinacea* Shreb. (Table 3). These results can likely be explained with consideration that Zn pseudo-total content and soluble concentration in soil were higher in F respect to S, and that the uptake of Zn has been reported to be linear with PTE concentration in soils (Kabata-Pendias, 2011). Zinc plays essential role in plant metabolism (Kabata-Pendias, 2011), so the mean effect of the compost application decreased Zn content (Table 3) probably due to a dilution effect caused by the greatly increased yields or vegetative growth (Zhou et al., 2012). Wu et al. (2011) reported similar results with compost application showing lower Zn plant content in mine soils.

### Microbial indicators of soil health under phytoremediation

Monitoring of the abundance of nitrogen cycling bacteria, as N-fixing and ammonia-oxidizing bacteria (Fig. 2), clearly showed differences between the compared substrates. The concentration of these bacterial groups recorded in F substrate were significantly lower than S, supporting the hypothesis linked to the lower biomass production in soil post-washing sludges attributed to a low activity of N-cycling microbes. This effect could be due to the tendency to higher Zn and Pb bioavailable fraction in F substrate than S as reported above. In fact, these bacterial functional groups, and in particular nitrifying bacteria, are considered one of the most sensitive to PTEs since even small traces of Cd, Ni, Cr, Cu, and Zn are toxic and limit their survival (Broos et al., 2005; Cela & Sumner, 2002; Chandran & Love, 2008; You et al., 2009).

Soil fertilization and plant inoculation differentially impacted N-cycling bacteria in the two substrates.

Compost addition significantly increased the abundance of *nif*H and *amo*A genes of both substrates, with a more marked effect in soil post-washing sludges, showing an overall pattern similar to crop dry-matter accumulation (Table 1). It is recognized that organic fertilization can stimulate N fixation and bacterial abundance (Kondo & Yasuda, 2003), and that compost amendments can aid in the promotion and restoration of multi-contaminated soil biofertility (Ventorino et al., 2019). In addition, compost could represent a new source of microorganisms that could help increase soil fertility (nitrogen-fixing and nitrifying bacteria), increase structural stability (exopolysaccharide producers), and synthetize hormones and nutrients, thus, increasing biological activity and soil quality (Boulter et al., 2002; Buyer et al., 2010).

However, the lower impact of compost on soil microbial populations in F than S substrate could be due to the different physical soil properties and in particular to the fine-textured and poorly aggregated substrates found in F that could strongly influenced the microorganisms (Poly et al., 2001).

Commercial biostimulants exerted a lower effect on the abundance of *nif*H and *amo*A genes in respect to compost. In fact, only TA significantly stimulated N-fixing and ammonia-oxidizing bacteria in S substrate and only in association with organic amendment. Bioinoculants could have noticeable effects on resident rhizospheric microflora stimulating their growth and activity (Chouyia et al., 2020; Gupta et al., 2016). The different influence of the two commercial bioinoculants could be due especially to their composition. Unlike TB, which only contains *T. harzianum* T22, TA consists of a combination of various mycorrhiza, *Bacillus*, and *Trichoderma* species with different biological activities that can influence plant growth promotion with diverse action mode. Moreover, the addition of compost to TA inoculated soils could also improve both the growth and the enzymatic activity of microbial populations in the rhizosphere (Sellamuthu & Govindaswamy, 2003). Although the influence of humic substances on soil microbiota depends on the type and source of the organic component (Suárez-Estrella et al., 2008), their effect could be due to mineral nutrients increased surfactant-like absorption (Bünemann et al., 2006). However, the microbes present in TA treatment seem to be stimulated only in the presence of compost demonstrating that organic fertilization coupled with TA inoculation could represent a viable strategy to restore microbial fertility of some PTE-contaminated industrial soils.

### Potentially toxic elements transfer from soil to humans via soil ingestion, inhalation, and dermal contact

A higher PTE bioaccessibility via ingestion and inhalation was reported than dermal bioaccessibility (Table 4), essentially because of the very acidic pH (1.5) of SBET formulation and the long duration of ALF extraction (24 h, i.e., PM_10_ and PM_2.5_ average residence time in the human respiratory system before the complete mucociliary clearance; Kastury et al., 2017). Besides pH and time, PTE bioaccessibility was strongly regulated by the biochemical composition of the synthetic fluids, consisting of salts, organic compounds, and enzymes, which may have led to PTE complexation, precipitation, etc. As well, the physico-chemical properties of the S and F samples and the origin and chemical species of the contaminants had a key role in the PTE bioaccessibility rates.

The bioaccessibility tests extracted higher bioaccessible contents of PTEs from fine (< 2.5 and 2.5–10 µm) than coarse (10–20 and 20–50 µm) particle-size fractions (Table 4); this may be justified by the higher surface area to volume ratio and PTE adsorbing capacity of the small particles, and maybe to their lower biodurability within body fluids (Lollar, 2005). Further, it can also depend on the increase in organic matter, Fe/Al oxides, and clay minerals (Li et al., 2020). An analogous trend was observed in other studies, assessing PTE bioaccessibility in particles of different sizes (Khelifi et al., 2021; Midander et al., 2007).

The monitoring of PTE bioaccessibility throughout the plant experiment unveiled a slight reduction (not statistically significant) of PTE bioaccessibility due to plant growth combined to substrate (bio)-fertilization (Table 4); the improved physico-chemical interactions among S and F minerals, humic substances and PTEs, and plant growth-promoting effect induced by compost (Table 1), associated to the enhanced uptake of most bioavailable PTE fractions by turfgrass (Table 3), could explain, at least in part, the overall lower bioaccessibility of PTEs. This indicates that the assisted grass-based phytoremediation system could have positive outcomes on the reduction of the environmental and health risks of the study site. Moreover, a permanent green capping of the area can limit the wind erosion and dispersion of health-risky particulate (10 µm) in the atmosphere.

Other than the bioaccessible contents of PTEs, serious non-carcinogenic and carcinogenic risks for human health, especially in children are most likely due to the ingestion of As- and Pb-enriched soil particles as revealed by US.EPA-based risk assessment (Table 5). These worrisome results were largely affected by the parameters associated to each exposure route (US.EPA, 2011) and the PTE-specific reference values (related to the toxicity of each PTE). In other words, the real risks to human health are probably lower than those estimated by risk assessment (Table 5), if taken into account the nature, geochemical distribution and scarce mobility of these PTE (As and Pb, in particular) in the study area, as widely ascertained by Adamo et al., (2002, 2015) and Rocco et al. (2018). Moreover, the negative impact of studied soil and sludges on human health exists only when they are not covered by the grass vegetation. These results ones again highlight the important role that a stable grass plant covering can play reducing the risks associated to the dispersion of soil contaminants.

## Conclusions

An important consideration regarding the management of industrial contaminated sites located close to residential areas, and their conversion to urban parks providing green open spaces to citizens, is the development of a widespread operative protocol to stabilize low-mobility pollutants (i.e., Rhur industrial region), thus, ensuring a successful recovery of the site and having a positive impact on public wellbeing. The aim of our study was to evaluate the effects of organic amendment and biostimulants on the phytostabilization potential of a vegetative capping by using a commercial grass mix to test in this hypothesis. The selected plant species were well adapted to the contamination levels found in the ILVA brownfield pre-washing soil and post-washing sludges, demonstrating a growth potential comparable to that of non-contaminated soils. In addition, soil covering was always above 90%, thus, sustaining the hypothesis that this grass-based phytoremediation system can limit movement of contaminated particulate suspensions, thus, minimizing the risks for human health via soil ingestion, inhalation, and dermal contact. Both compost fertilization and inoculations with the biostimulant microbial consortium containing mycorrhiza, plant growth-promoting bacteria, and *Trichoderma* species, increased the plant growth, thus, soil covering, providing a viable strategy to increase turfgrass density and, at the same time, lightly reducing the bioaccessibility of main PTEs. The growth and the abundance of N_2_-fixing and ammonia-oxidizing bacteria increased significantly, particularly in the combined CTA treatment demonstrating that these strategies are also able to restore soil biological fertility, which is usually the main constraint in phytoremediation of degraded soils. Nevertheless, a mid- to long-term period is required to recover, renew, and maintain contaminated soils in comparison to natural ecosystems, since soil contamination is often associated to physical problems of poorly structured fine soils. As indicated in our experiment with the grass-based phytoremediation system established on industrial soils, the lead content in the examined grass tissues was below the legal threshold indicated for forage crops, suggesting that there will be no risks associated to the accidental intake of this toxic compound in the plant biomass by fauna.

Obviously, it must be pointed out that, even if very low PTE translocation occurs in shoot biomass, the management of the potentially contaminated sites should exclude the presence of grazing animals due to the ever-present possibility that there is ingestion of the vegetation with adhering contaminated soil particles.

## Supplementary Information

Below is the link to the electronic supplementary material.

Supplementary file1 (DOCX 29 kb)

## Data Availability

Data are available from the authors if needed for review.
