# Supplementary material for: Compost and microbial biostimulant applications improve plant growth and soil biological fertility of a grass-based phytostabilization system
Source: Environ Geochem Health. 2022 Mar 22;45(3):787–807. doi: 10.1007/s10653-022-01235-7 (PMC10014777; doi:10.1007/s10653-022-01235-7)
Supplement: Supplementary file 1 — Supplementary file1 (DOCX 29 kb) [file 10653_2022_1235_MOESM1_ESM.docx]

**SUPPLEMENTARY MATERIAL**

**Compost and microbial biostimulant applications improve plant growth and soil biological fertility of a grass-based phytostabilization system**

Donato Visconti*^1^, Valeria Ventorino^1^, Massimo Fagnano^1^, Sheridan Lois Woo^2,3,4^, Olimpia Pepe^1^, Paola Adamo^1^, Antonio Giandonato Caporale^1^, Linda Carrino^1^, Nunzio Fiorentino^1,3^

^1^ Department of Agricultural Sciences, University of Naples Federico II, 80055 Portici, Italy

^2^ Department of Pharmacy, University of Naples Federico II, 80131 Naples, Italy

^3^Center for studies on Bioinspired Agro-environmental Technology (BAT Center), 80055 Portici, Italy

^4^ National Research Council, Institute for Sustainable Plant Protection, 80055 Portici, Italy

*Corresponding author: donato.visconti@unina.it

**Table S1** Physico-chemical properties and PTE pseudo-total contents in sludge (F) and soil (S) samples.

|  |  | **Sludge (F)** | **Soil (S)** | **Screening Values^a^** | |
| --- | --- | --- | --- | --- | --- |
|  |  |  |  | residential site | industrial site |
| Sand | % | 30 | 65 |  |  |
| Silt | % | 62 | 33 |  |  |
| Clay | % | 8 | 2 |  |  |
| pH |  | 7.6 | 7.5 |  |  |
| EC | μS cm^-1^ | 358 | 322 |  |  |
| Carbonates | % | 3.7 | 5.3 |  |  |
| OM | g kg^-1^ | 24.8 | 19.5 |  |  |
| OC | g kg^-1^ | 14.4 | 11.3 |  |  |
| TN | g kg^-1^ | 0.9 | 0.7 |  |  |
| Cu | mg kg^-1^ | 69 | 69 | 120 | 600 |
| Pb | mg kg^-1^ | **281** | **171** | 100 | 1000 |
| Zn | mg kg^-1^ | **1208** | **362** | 150 | 1500 |
| Ni | mg kg^-1^ | 21 | 72 | 120 | 500 |
| Co | mg kg^-1^ | 12 | 14 | 20 | 250 |
| As | mg kg^-1^ | **47** | **41** | 20 | 50 |
| Cd | mg kg^-1^ | 1.9 | 1.1 | 2 | 15 |
| Sb | mg kg^-1^ | 9.9 | 6.7 | 10 | 30 |
| Cr | mg kg^-1^ | 28 | 143 | 150 | 800 |

^a^ Screening values by Italian Law Decree 152, 2006. In bold, values greater than the acceptable Italian screening values.

**Table S2** Substrate mean effect on $E_{r}^{i}$ and ERI.

| Treatments | $E_{r}^{i}$ As | $E_{r}^{i}$ Cd | $E_{r}^{i}$ Pb | $E_{r}^{i}$ Zn | ERI |
| --- | --- | --- | --- | --- | --- |
| **Substrate** |  |  |  |  |  |
| F | 38.47 **a** | 65.00 **a** | 27.61 **a** | 16.9 **a** | 148 **a** |
| S | 33.61 **b** | 31.67 **b** | 18.78 **b** | 5.6 **b** | 89 **b** |
| *Significance* | ** | ** | ** | ** | ** |

F and S are sludge and soil substrates, respectively. Mean values with the same letter do not differ according to the LSD test (p<0.05). *p<0.05; **p<0.01; n.s., not significant.

**Fig. S1** Compost effect on Pb concentrations in meadow aboveground tissues: substrate by fertilization interaction. C and NoC are compost fertilized and non-fertilized treatments, respectively; F and S are soil washing sludge and pre-washing soil, respectively. Bars indicate ± standard errors. Mean values with the same letter do not differ according to the LSD test (p<0.05).
